# Aprepitant attenuates NLRC4-dependent neuronal pyroptosis via NK1R/PKCδ pathway in a mouse model of intracerebral hemorrhage

**DOI:** 10.1186/s12974-022-02558-z

**Published:** 2022-08-03

**Authors:** Peng Jin, Dongqing Qi, Yuhui Cui, Cameron Lenahan, John H. Zhang, Xiaogen Tao, Shuixiang Deng, Jiping Tang

**Affiliations:** 1 Department of Intensive Care Unit, The First Affiliated Hospital of USTC, Division of Life Sciences and Medicine, University of Science and Technology of China, Hefei, 230001 Anhui China; 2 Department of Physiology and Pharmacology, Loma Linda University, Risley Hall, Room 219, 11041 Campus Street, Loma Linda, CA 92354 USA; 3 Department of Rehabilitation Medicine, The First Affiliated Hospital of USTC, Division of Life Sciences and Medicine, University of Science and Technology of China, Hefei, 230001 Anhui China; 4 Department of Neurosurgery, Loma Linda University, Loma Linda, CA 92350 USA; 5 Burrell College of Osteopathic Medicine, Las Cruces, NM 88001 USA; 6 Department of Anesthesiology, Loma Linda University, Loma Linda, CA 92350 USA; 7 Department of Intensive Care Unit, Huashan Hospital, Fudan University, 12 Urumqi Road, Shanghai, 200040 China; 8 Department of Neurosurgery, Sixth People’s Hospital Affiliated to Shanghai Jiao Tong University, Shanghai, 200040 China

**Keywords:** Aprepitant, Intracerebral hemorrhage, Inflammasome, Neurokinin receptor 1, Pyroptosis

## Abstract

**Background:**

Pyroptosis is a programmed cell death mediated by inflammasomes. Previous studies have reported that inhibition of neurokinin receptor 1 (NK1R) exerted neuroprotection in several neurological diseases. Herein, we have investigated the role of NK1R receptor inhibition using Aprepitant to attenuate NLRC4-dependent neuronal pyroptosis after intracerebral hemorrhage (ICH), as well as the underlying mechanism.

**Methods:**

A total of 182 CD-1 mice were used. ICH was induced by injection of autologous blood into the right basal ganglia. Aprepitant, a selective antagonist of NK1R, was injected intraperitoneally at 1 h after ICH. To explore the underlying mechanism, NK1R agonist, GR73632, and protein kinase C delta (PKCδ) agonist, phorbol 12-myristate 13-acetate (PMA), were injected intracerebroventricularly at 1 h after ICH induction, and small interfering ribonucleic acid (siRNA) for NLRC4 was administered via intracerebroventricular injection at 48 h before ICH induction, respectively. Neurobehavioral tests, western blot, and immunofluorescence staining were performed.

**Results:**

The expression of endogenous NK1R and NLRC 4 were gradually increased after ICH. NK1R was expressed on neurons. Aprepitant significantly improved the short- and long-term neurobehavioral deficits after ICH, which was accompanied with decreased neuronal pyroptosis, as well as decreased expression of NLRC4, Cleaved-caspase-1, GSDMD (gasdermin D), IL-1β, and IL-18. Activation of NK1R or PKCδ abolished these neuroprotective effects of Aprepitant after ICH. Similarly, knocking down NLRC4 using siRNA produced similar neuroprotective effects.

**Conclusion:**

Aprepitant suppressed NLRC4-dependent neuronal pyroptosis and improved neurological function, possibly mediated by inhibition of NK1R/PKCδ signaling pathways after ICH. The NK1R may be a promising therapeutic target for the treatment of ICH.

**Supplementary Information:**

The online version contains supplementary material available at 10.1186/s12974-022-02558-z.

## Introduction

Intracerebral hemorrhage (ICH) is a devastating subtype of stroke, accounting for approximately 10–15% of all reported stroke cases worldwide each year [1, 2]. Moreover, it is associated with higher mortality and long-term disability compared with ischemic stroke [3]. Brain injury after ICH is mediated by multiple pathophysiological mechanisms that can be broadly classified into primary and secondary brain injury (SBI) [4]. Initial hematoma volume and expansion are two key factors in primary injury after ICH [5]. However, to date, surgical removal of the hematoma and various therapeutic interventions to inhibit hematoma expansion have not been proven to be effective for ICH patients according to clinical evidence [6, 7]. Conversely, increasing evidence has shown that secondary brain injury, including inflammatory response, oxidative stress, and apoptosis caused by various adverse factors, is closely related to the outcome of ICH [8]. However, to date, the specific molecular mechanisms of neuronal death remain poorly understood.

Pyroptosis, a programmed cell death mediated by inflammasomes and characterized by the opening of cell membrane pores which release inflammatory cytokines into the surrounding environment, is identified as an important mechanism of inflammation-induced neuronal cell death in a variety of neurological diseases [9–11]. After activation, inflammasomes convert precursor caspase-1 to cleaved caspase-1, which further converts precursor IL-1β and IL-18 into biologically active mature pro-inflammatory IL-1β and IL-18, respectively, which are then released from cell membrane pores, thereby recruiting more inflammatory cells and inducing the cascade inflammatory response [12, 13]. Inflammasomes found in brain include NLRP1 (NLR Family Pyrin Domain Containing 1), NLRP3 (NLR Family Pyrin Domain Containing 3), NLRC4 (NLR Family CARD Domain Containing 4), and AIM2 (Absent. In Melanoma 2) [14]. Moreover, previous studies have confirmed that the neurological function in mice with ICH can be significantly improved by inhibiting NLRP1- and NLRP3-mediated neuronal pyroptosis [15, 16]. However, to date, no study has confirmed whether NLRC4-mediated pyroptosis exerts the same therapeutic effect.

Substance P (SP) is a neuropeptide that is widely distributed throughout the central nervous system (CNS) and is actively involved in inflammatory processes [17]. Lorente et al. showed that serum substance P levels were strongly associated with mortality in patients with cerebral hemorrhage [18]. SP exerts its biological effects by binding to the family of neurokinin receptors (NKRs). Among which, it has a high affinity for NK1R, a seven transmembrane domain G protein-coupled receptor (GPCR) expressed by microglia, neurons, and astrocytes [19]. Studies have shown that inhibition of SP/NK1R can exert neuroprotective effects, such as anti-inflammatory, antioxidant, and anti-apoptotic effects in a variety of neurological diseases [19]. In our previous study, we demonstrated that Aprepitant (chemical name: 3- [ [(2S,3R)-2- [(1R)-1- [3,5-bis(trifluoromethyl)phenyl]ethoxy]-3-(4-fluorophenyl)morpholin-4-yl]methyl]-1,4-dihydro-1,2,4-triazol-5-one), a specific inhibitor of NK1R, reduces hematoma volume and thus improves neurological deficits in ICH mice [20].

The binding of SP to NK1R activates phospholipase C (PLC), leading to the formation of diacylglycerol, which activates protein kinase C (PKC) [21]. Koh et al. demonstrated that inhibition of NK1R reduced PKCδ phosphorylation in murine pancreatic acinar cells [22], and Wang et al. similarly demonstrated that in microglia, activation of NK1R increased the phosphorylation level of PKCδ [23]. Previous studies have shown that intracellular levels of NLRC4 are mainly regulated by phosphorylated PKCδ [24, 25].

In the current study, we hypothesized that NK1R activation promoted NLRC4-mediated pyroptosis after ICH. Aprepitant would attenuate neurological deficits and decrease neuronal pyroptosis through the NK1R/PKCδ signaling pathway after ICH in mice.

## Materials and methods

### Animals

All experimental procedures involving animals in this study were conducted in accordance with the National Institutes of Health (NIH) Guide for the Care and Use of Laboratory Animals and were approved by the Institutional Animal Care and Use Committee (IACUC) of Loma Linda University. Adult male mice (8–12 weeks) were used in this study. They were housed in a controlled humidity and temperature room with a 12-h light and dark cycle.

### Experiment design

**Fig. 1:**
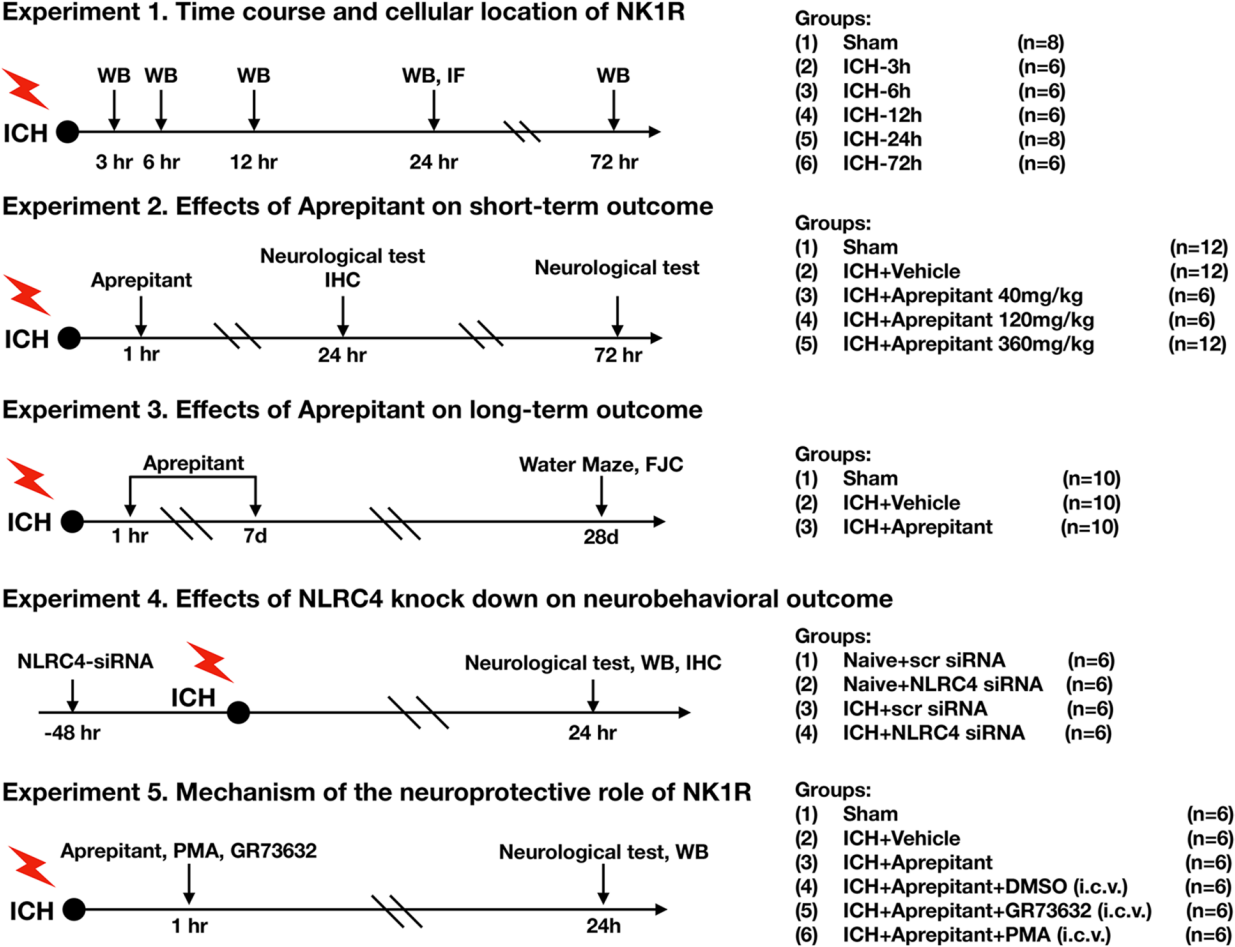
Experimental design and animal groups. ICH, intracerebral hemorrhage; WB, western blot; IHC, immunohistochemistry; DMSO, dimethyl sulfoxide; i.p., intraperitoneal; i.c.v., intracerebroventricularly

Five separated experiments were performed as follows (Fig. 1).

#### Experiment 1

To determine the time course of endogenous NK1R and NLRC4 expression after ICH, 36 mice were randomly divided into 6 groups: 0 (sham), 3 h, 6 h, 12 h, 24 h, 72 h (*n* = 6/group) after ICH induction for western blot analysis to measure NK1R and NLRC4 expression in the ipsilateral hemisphere. In addition, 4 mice were randomly divided into sham (*n* = 2) and ICH-24 h (*n* = 2) for double immunofluorescence to demonstrate the co-localization of NK1R with neurons.

#### Experiment 2

To evaluate the role of NK1R inhibition by Aprepitant in the post-ICH brain injury. 30 mice were randomly assigned to either sham, ICH + vehicle, or ICH + Aprepitant (40, 120, 360 mg/kg). Modified Garcia, forelimb placement, and corner turn tests were performed to assess neurological function at 24 and 72 h after ICH. Based on the results of neurobehavioral tests, 18 mice were divided into sham, ICH + vehicle, and ICH + Aprepitant (best dose) for western blot, FJC, and TUNEL staining at 24 h after ICH.

#### Experiment 3

To investigate the effect of Aprepitant on long-term outcomes after ICH, 30 mice were randomly assigned to three groups: sham, ICH + vehicle, and ICH + Aprepitant (120 mg/kg) (*n* = 10/group). Aprepitant was administered daily by intraperitoneal injection from day 1 to day 7 after surgery. Rotarod and foot faults tests were performed on weeks 1, 2, and 3 after ICH induction. Morris water maze tests were performed on days 23–28 after ICH. After that, the mice were euthanized and subjected to FJC staining to assess neuronal degeneration.

#### Experiment 4

NLRC4 siRNA was used explore the role of NLRC4 on neurological deficits and neuronal pyroptosis after ICH. Twenty-four mice were randomly assigned to the following groups (*n* = 6/group): naïve + scr siRNA, naïve + NLRC4 siRNA, ICH + scr siRNA, and ICH + NLRC4 siRNA. Neurobehavioral tests were performed 24 h after ICH and western blot was used to assess protein levels of NLRC4 and C-Caspase-1 in the ipsilateral hemisphere brain tissue.

#### Experiment 5

To assess the underlying mechanism of NK1R/PKCδ in NLRC4-dependent neuronal pyroptosis. A total of 36 mice were randomized into the following 6 groups (n = 6/group): sham, ICH + vehicle, ICH + Aprepitant, ICH + Aprepitant + DMSO, ICH + Aprepitant + GR73632, and ICH + Aprepitant + PMA. GR73632 or PMA was administered via i.c.v injection 1 h after ICH. Neurobehavioral tests and Western blots were conducted at 24 h after ICH.

### Intracerebral hemorrhage model

The ICH model was induced by autologous blood injection according to previous studies [26, 27]. Briefly, the mice were anesthetized via intraperitoneal injection of ketamine (100 mg/kg) and xylazine (10 mg/kg), and then placed on a stereotaxic frame. A total of 30 μL of blood taken from the left femoral artery was injected into the right basal ganglion (coordinates: 0.2 mm anterior, 2.2 mm lateral and 3.5 mm ventral to the bregma) through a Hamilton syringe at a rate of 3 ul/min. The needle remained in place for 10 min, then slowly withdrawn, and the surgical incision was sutured after sealing the skull burr hole with bone wax. The mice were closely monitored until full recovery from anesthesia. Sham surgery was performed following the same procedure without the whole blood infusion.

### Short-term neurological outcomes assessment

The Modified Garcia, forelimb placement, and corner turn tests were performed to evaluate short-term neurological functions as previously described [28, 29]. Modified Garcia test was conducted to assess spontaneous activity, axial sensation, tactile proprioception, limb motor symmetry, lateral bending, forelimb walking, and climbing. With a maximum score of 21, lower scores indicated more pronounced neurological deficits. The forelimb placement test was performed by holding the mouse's trunk and approaching the platform to touch its left tentacle. Results were recorded as the percentage of left forelimb placements on the platform. For the corner turn test, the mice were allowed to advance into a 30° corner. The number of turns to the left or right was recorded and repeated 10 times. The percentage of left turns was calculated.

### Long-term neurological outcomes assessment

As previously described [30], to assess spatial learning ability and memory, the Morris water maze test was performed on days 23–28 after ICH. Briefly, the mice were placed in a semi-random starting position to start searching for a visible platform above the horizontal plane. If the mice did not find the platform within 60 s, they were manually guided to the platform and remained on the platform for 5 s. The swimming paths, distances, and times to reach the platform were recorded. On the last day, the platform was removed for the probe test and the time spent in the probe quadrant was recorded.

Foot fault test and rotarod test were performed as previously described [31]. In the foot fault test, mice moved along a horizontal grid (20 cm × 100 cm) for 2 min. The total number of the left forelimb missteps were recorded. In rotarod test, mice were placed on an accelerated rotating horizontal cylinder at a starting speed of 5 rpm with a regular increase in speed. Three trials were performed and the mean time of falling latency was recorded.

### FJC staining

Fluoro-Jade C (FJC) staining was conducted for assessing degenerated neurons as previously reported [32]. Briefly, the slides were first immersed in 1% sodium hydroxide solution for 5 min, followed by rinsing with 70% ethanol and distilled water for 2 min. Next, the slides were soaked in 0.06% potassium permanganate solution for 10 min and then rinsed with distilled water for 2 min. Then, the slides were incubated with 0.0001% FJC solution (Biosensis, USA) for 10 min. The FJC-positive cells were counted in six sections per mouse in a blinded manner with a fluorescence microscope. Data were expressed as the average number of FJC-positive cells/mm^2^ in the peri-hematomal regions and ipsilateral hippocampus regions.

### TUNEL staining

Double staining of NeuN and terminal deoxynucleotidyl transferase dUTP nick end labeling (TUNEL) was conducted using the Apoptosis Detection Kit (Roche, USA) according to the manufacturer's instructions for quantifying neuronal apoptosis surrounding the hematoma 24 h after ICH [33]. The number of TUNEL-positive neurons was counted manually at × 200 magnification in the peri-hematoma region. Data were expressed as the ratio of TUNEL-positive neurons (%).

### Intracerebroventricular administration

GR73632 (Bio-Techne Corporation, USA), PMA (Sigma Aldrich, USA), NLRC4 siRNA (Thermo Fisher Scientific, USA) or scr siRNA was given by intracerebroventricular injection as previously described [34]. Briefly, under isoflurane anesthesia, mice were placed in a stereotaxic frame. A 26-gauge needle of a 10-μl Hamilton syringe was inserted through the cranial burr hole into the left ventricle with the following coordinates relative to the bregma: 0.3 mm posterior, 1.0 mm lateral, and 2.3 mm deep. The rate was controlled at 1 μL/min using an infusion pump (Harvard Apparatus, USA). The needle remained in place for 5 min after injection and was then retracted slowly over a period of 5 min.

### Western blot

Western blotting was performed as previously described [35]. Briefly, the mice were deeply anesthetized with isoflurane, followed by intracardial perfusion with iced PBS. The ipsilateral hemispheres were separated, frozen with liquid nitrogen, and then stored in a – 80 °C freezer until use. Samples were homogenized in RIPA lysis buffer (Santa Cruz Biotechnology, USA) with a protease inhibitor for 15 min and centrifuged at 14,000*g* (4 °C, 30 min), followed by supernatant collection. Protein concentration in the supernatants was measured using a detergent-compatible assay (DC Protein Assay, Bio-Rad Laboratories, USA). Equal amounts of proteins were loaded onto SDS-PAGE gels, run by electrophoresis, and then transferred to nitrocellulose membranes. The membrane was blocked with 5% nonfat milk (Bio-Rad Laboratories, USA) for 2 h and incubated overnight at 4 °C with the following primary antibodies: anti-NK1R (1:500, Santa Cruz Biotechnology, USA); anti-PKCδ (1:2000, Santa Cruz Biotechnology, USA); anti-p-PKCδ (1:500, Santa Cruz Biotechnology, USA); anti-NLRC4 (1:1000, Cell Signaling Technology, USA); anti‐caspase‐1 (1:500, Novus, USA), anti‐IL‐1β (1:1000, Abcam, USA), and anti-β-actin (1:4000, Santa Cruz Biotechnology, USA). The following day, the appropriate secondary antibody (1:3000, Santa Cruz Biotechnology, USA) was selected to incubate membranes at room temperature on the following day. Immunoblots were then visualized using an ECL Plus chemiluminescence reagent kit (Amersham Bioscience, USA). The band density was quantified using Image J (NIH, Bethesda, USA). The results were standardized using β-actin as an internal control.

### Immunofluorescence staining

Double-immunofluorescence staining was conducted as previously described [32]. Mice were perfused intracardially with PBS and formalin. The brain samples were soaked in formalin for 1 day and then dehydrated by sucrose solutions. Samples were frozen in OCT and sliced into 10-µm-thick coronal sections using a cryostat (CM3050S; Leica Microsystems, Germany). After being washed 3 times with 0.01 M PBS for 10 min each, the brain sections were incubated with 5% donkey serum at room temperature for 2 h and then incubated overnight at 4 °C with primary antibodies including: anti-NK1R (1:50, Santa Cruz Biotechnology, USA); anti-NeuN (1:100, Abcam, USA). The next day, the slices were incubated with fluorescence-conjugated secondary antibodies (1:500, Jackson Immuno Research) for 1 h at room temperature followed by visualization using a fluorescence microscope (Leica Microsystems, Germany).

### Statistical analysis

Data analysis was performed with GraphPad Prism (Graph Pad Software, USA). All data were expressed as the mean and standard deviation (mean ± SD). Two-way repeated-measures ANOVA followed by Tukey’s post hoc test was used to analyze the long-term neurological functions. Comparisons among groups were performed with one-way ANOVA followed by Tukey’s post hoc test. Statistical significance was defined as *p* < 0.05.

## Results

### Animal mortality and exclusion

A total of 182 mice were used in this study. Of which, 146 underwent ICH induction. The overall mortality rate of ICH mice was 6.16% (9/146), and the causes of death included complications from anesthesia, large amounts of blood into the ventricles, etc. There were no deaths in sham or naïve groups. Seven mice were excluded because of the absence of hemorrhage. In addition, 12 mice were shared in different experiments according to the "3Rs" (Replacement, Reduction, and Refinement) principle.

### Time course of endogenous protein levels of NK1R, NLRC4 in ipsilateral hemisphere (right) after ICH

**Fig. 2:**
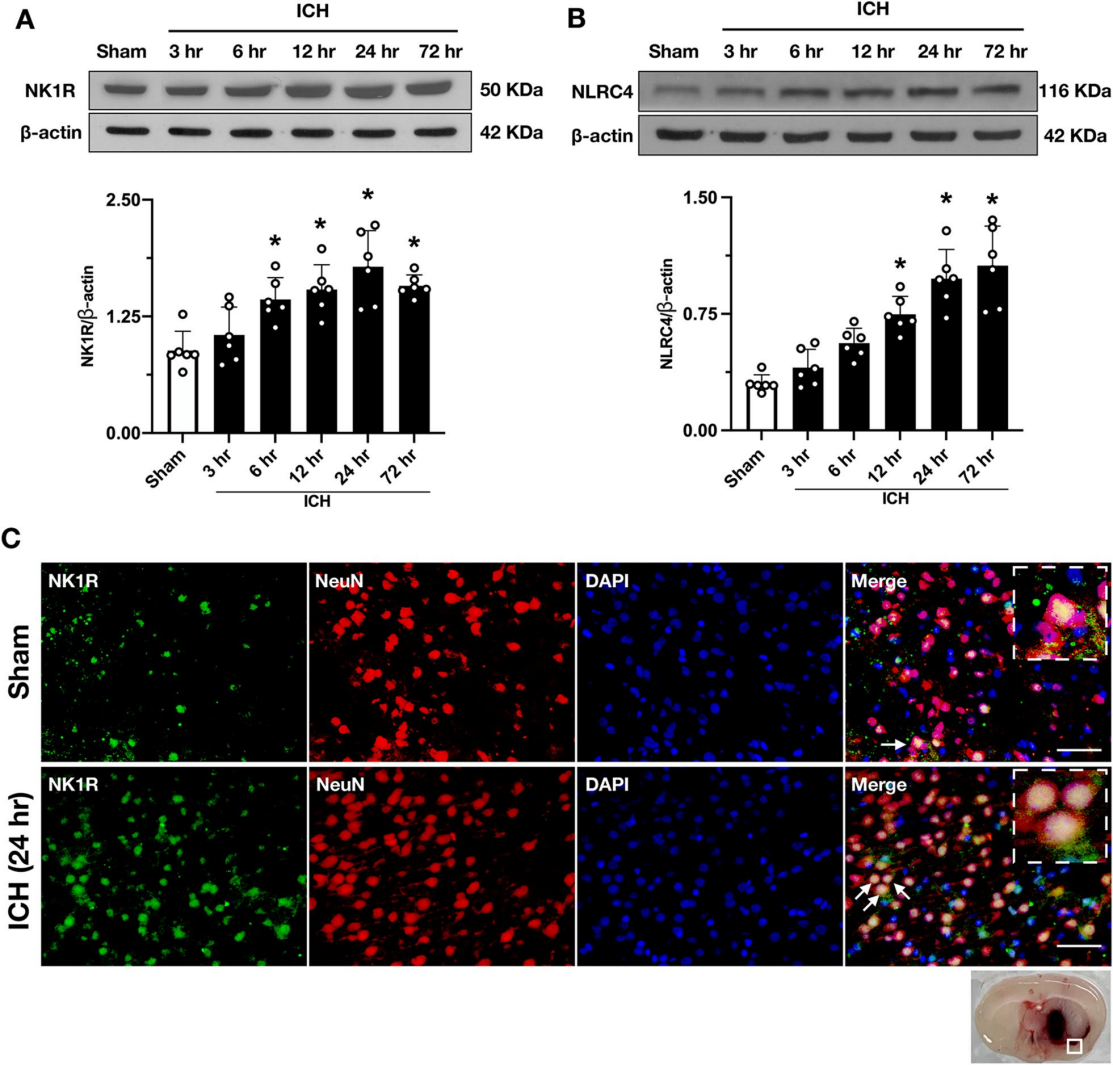
Time course of NK1R and NLRC4 protein levels after ICH, and cellular location of NK1R after ICH. **A** Representative western blot bands, and quantitative analyses of NK1R in the ipsilateral hemisphere after ICH. **B** Representative western blot bands, and quantitative analyses of NLRC4 in the ipsilateral hemisphere after ICH. Data were represented as mean ± SD. * *p* < 0.05 vs sham group; one-way ANOVA, Tukey test, *n* = 6/group. **C** Representative pictures of double immunofluorescence staining showed NK1R (green) co-localized with neurons (NeuN, red) in sham group and the peri-hematomal area 24 h after ICH. Scale bar = 50 μm. *n* = 2/group

The expression of endogenous NK1R and NLRC4 in the right cerebral hemisphere was assessed by Western blot. The results showed that the expression of NK1R was significantly increased at 6 h and peaked at 24 h after ICH when compared with sham group (*p* < 0.05, Fig. 2A). NLRC4 expression was similarly significantly upregulated and remained at peak levels from 24 to 72 h after ICH (P < 0.05, Fig. 2B). Double-immunofluorescence staining showed co-localization of NK1R with neurons. The number of NK1R-positive neurons in the peri-hematomal area was significantly increased at 24 h after ICH compared with sham group (Fig. 2C). Similarly, we found that the protein expression level of NK1R in the hippocampal region was significantly upregulated and the number of NK1R-positive neurons was increased after ICH (Additional file 1: Fig. S1A–C).

### Aprepitant treatment improved neurological deficits at 24 and 72 h after ICH

**Fig. 3:**
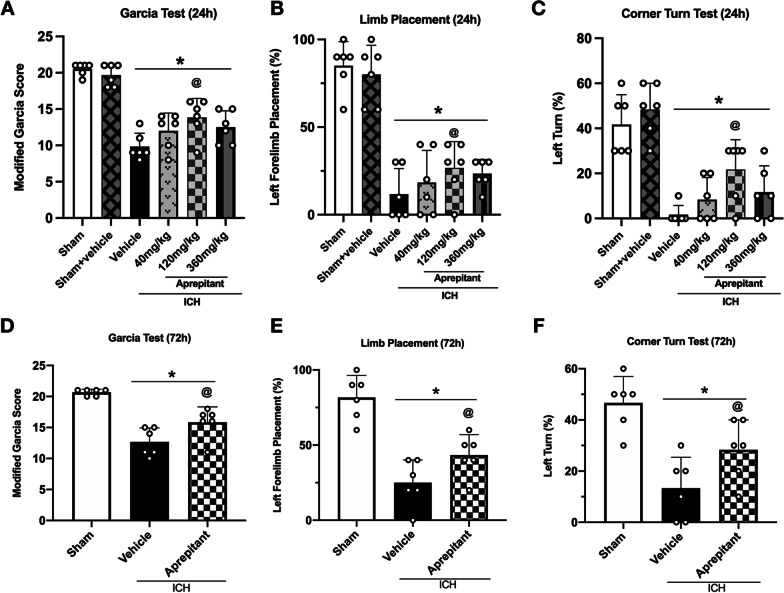
The effects of Aprepitant administration on neurobehavioral tests at 24 and 72 h post-ICH. **A**–**C** Modified Garcia test, forelimb placement test, corner turn test at 24 h after ICH. **D**–**F** Modified Garcia test, forelimb placement test, corner turn test at 72 h after ICH. Data were represented as mean ± SD. * *p* < 0.05 vs sham; @ *p* < 0.05 vs ICH + vehicle. One-way ANOVA, Tukey test, *n* = 6/group

Compared to the Sham group, mice in the Sham + Vehicle group did not exhibit worse neurological deficits, which confirms that intraperitoneal injection of DMSO does not have an effect on neurological function. However, mice in the ICH group showed significant neurological impairment at 24 and 72 h after surgery compared with sham group. To determine the optimal dose, three different (40, 120, 360 mg/kg) [36] doses of Aprepitant were administered intraperitoneally at 1 h after ICH to assess its efficacy on neurological outcome at 24 h. The results showed that the medium dose (120 mg/kg) significantly improved neurological deficits at 24 h after ICH (Fig. 3A–C). To further confirm the effectiveness of this dose, neurobehavioral tests were performed again at 72 h after ICH, and Aprepitant treatment still significantly improved the neurological deficits compared with ICH + Vehicle group (Fig. 3D–F). Based on these findings, the middle dose was selected for further study.

### Aprepitant attenuated neuronal pyroptotic cell death at 24 h after ICH

**Fig. 4:**
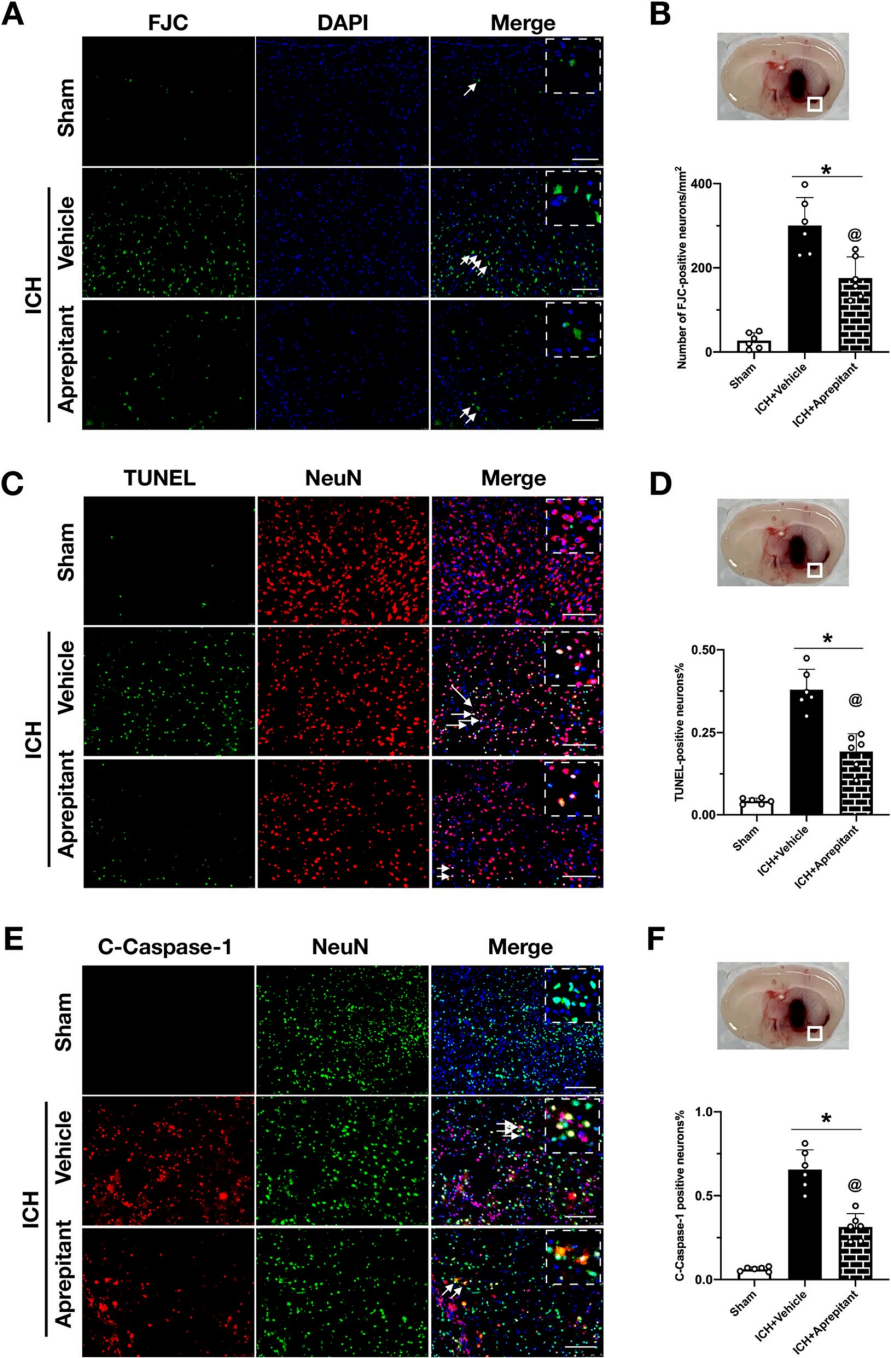
Effects of Aprepitant administration on neuronal degeneration and pyroptosis at 24 h post-ICH. **A**, **B** Representative images and quantitative analysis of Fluoro-Jade C (FJC) staining in the peri-hematomal area at 24 h after ICH. **C**, **D** Representative images and quantitative analysis of terminal deoxynucleotidyl transferase dUTP nick end labeling (TUNEL) staining in the peri-hematomal area at 24 h after ICH. **E**, **F** Representative images and quantitative analysis of cleaved caspase‐1 (C‐caspase‐1) positive neurons in the peri-hematomal area at 24 h after ICH. Data were represented as mean ± SD. * *p* < 0.05 vs sham; @ *p* < 0.05 vs ICH + vehicle. One-way ANOVA, Tukey test, *n* = 6/group. Scale bar = 200 µm

To assess neuronal damage, FJC and TUNEL stainings were performed. The results of FJC staining showed that the number of FJC-positive cells in the peri-hematomal area was significantly higher in the ICH + vehicle group compared with sham group. Moreover, Aprepitant treatment markedly reduced the number of FJC-positive cells, indicating that Aprepitant could significantly improve neuronal degeneration at 24 h after ICH (Fig. 4A, B). TUNEL staining presented with similar results. Aprepitant treatment also reduced the number of TUNEL-positive neurons (Fig. 4C, D). C-Caspase-1 is a specific marker of pyroptosis, and double immunofluorescence staining showed that Aprepitant significantly inhibited the increase of C-Caspase-1-positive neurons after ICH (Fig. 4E, F), indicating a protective effect of Aprepitant against neuronal pyroptosis.

### Aprepitant improved long-term neurological deficits at 4 weeks after ICH

**Fig. 5:**
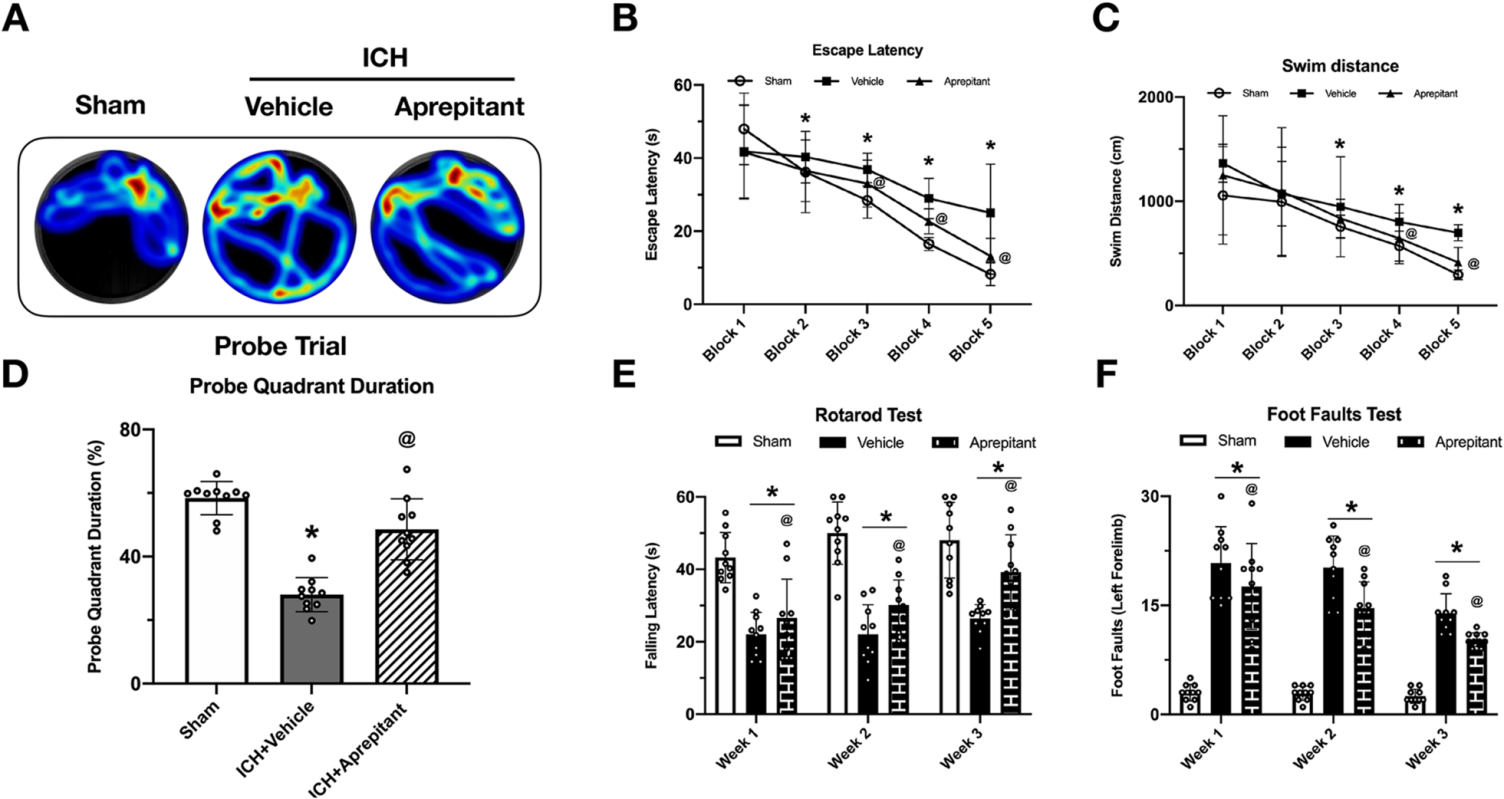
Effects of NK1R inhibition with Aprepitant on long-term outcomes at 28 d after ICH. **A** Representative heatmaps of the probe trial. **B**, **C** Escape latency and swim distance of Morris water maze on days 23 to 27 after ICH. **D** Probe quadrant duration on day 28 after ICH **E** Foot fault test and **F** rotarod test at day 7, 14 and 21 post-ICH. Data were represented as mean ± SD. *p < 0.05 vs sham; @P < 0.05 vs ICH + vehicle group; one-way ANOVA, Tukey post hoc test, *n* = 15/group

At 23–28 days after ICH, Morris water maze tests were performed to assess spatial memory and learning ability. Compared with the sham group, ICH mice had increased escape latency and swimming distance on days 25 to 27 after ICH. Surprisingly, Aprepitant treatment significantly shortened escape latency time on days 25 to 27, and reduced the swimming distance on days 26 and 27 (Fig. 5B, C). Similarly, in the probe trials on day 28 after ICH, Aprepitant could increase the time spent in the target quadrant (Fig. 5A, D). These results suggest that the impaired spatial memory and memory abilities of ICH mice were significantly improved after Aprepitant treatment.

Rotarod and foot fault tests were performed at weeks 1, 2, and 3 after ICH, and when compared with the sham group, the ICH mice had more foot faults and shorter falling latency in rotarod test. Aprepitant treatment significantly improved the behavioral performance in the foot fault and rotarod tests in the ICH group (Fig. 5E, F).

### Aprepitant reduced neuronal degeneration in hippocampus region at 28 days after ICH

**Fig. 6:**
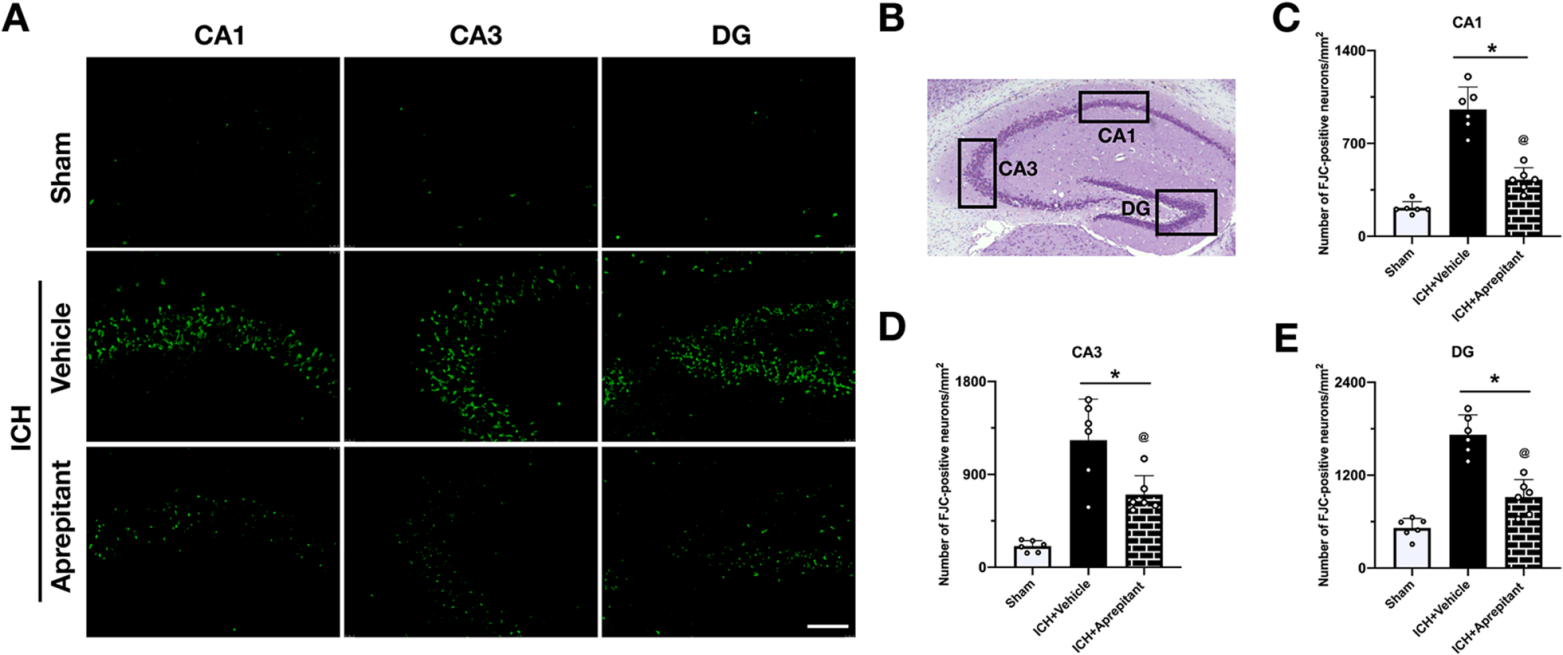
Effect of Aprepitant on neuronal degeneration at 28 days after ICH. **A** Representative Fluoro-Jade C staining pictures in different regions of hippocampus at 28 days after ICH. **B** Regions of interest areas in the ipsilateral hippocampus. Quantification of the Fluoro-Jade C-positive cells in **C** CA1, **D** CA3, and **E** DG region. Data were represented with mean ± SD. *P < 0.05 vs sham; @*P* < 0.05 vs ICH + vehicle group; one-way ANOVA, Tukey post hoc test, *n* = 6/group. Scale bar = 200 µm

Fluoro-Jade C staining was used to assess neuronal degeneration in the hippocampal region. A large number of degenerated neurons were observed in the ipsilateral CA1 regions at 28 days after ICH. However, Aprepitant treatment significantly reduced the number of Floro-Jade C-positive neurons in ipsilateral hippocampus when compared to ICH + vehicle group (Fig. 6A–E).

### NLRC4 siRNA decreased the expression of NLRC4 in naïve and ICH mice, and improved neurological deficits after ICH

**Fig. 7:**
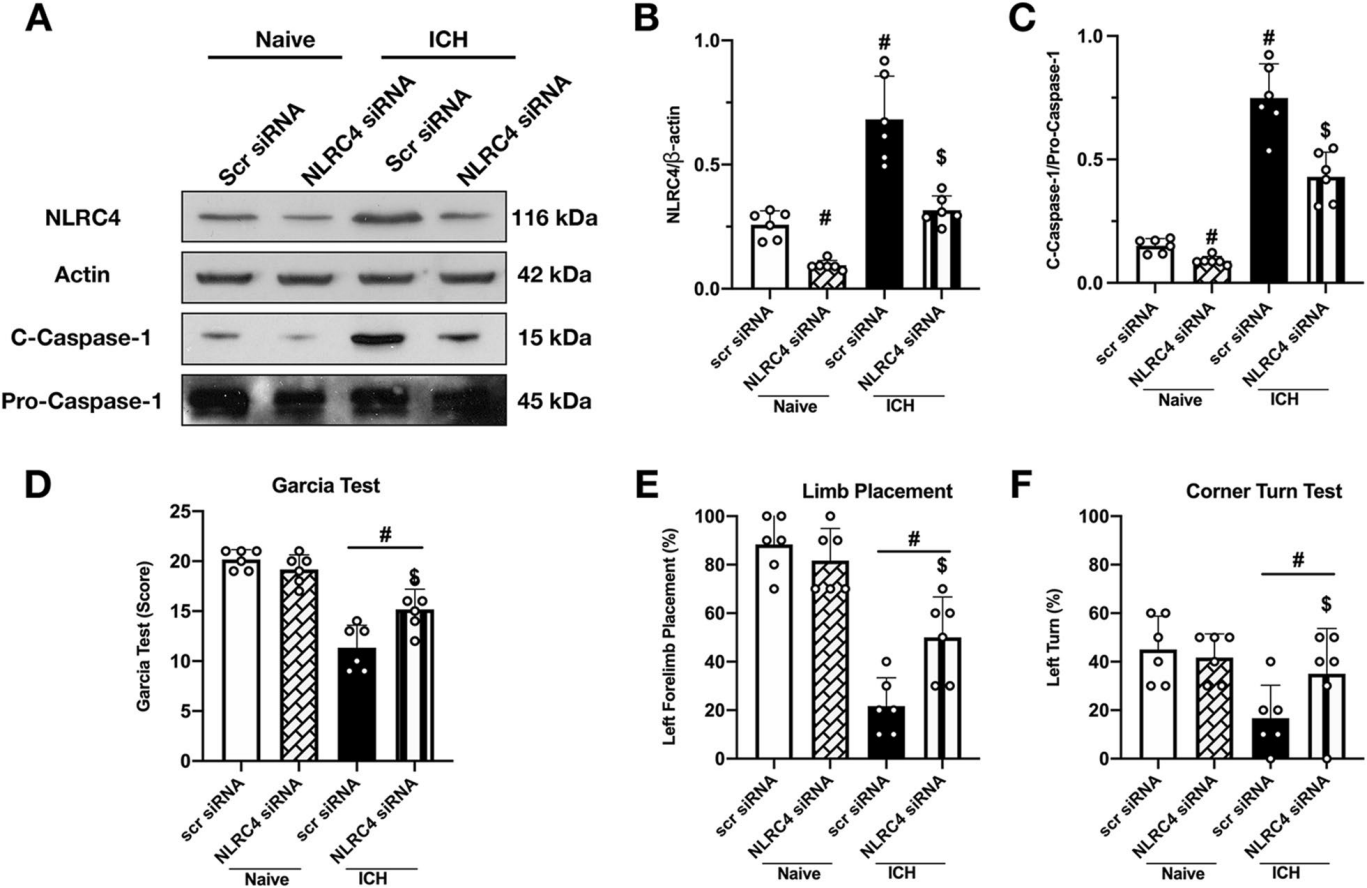
Effects of NLRC4 knock down with siRNA on the level of C-Caspase-1 and neurological function at 24 h after ICH. **A** Representative western blot bands of NLRC4 and C-Caspase-1 after administration of NLRC4 siRNA, and **B**, **C** quantitative analyses of NLRC4 and C-Caspse-1; **D**–**F** Modified Garcia test, forelimb placement test, corner turn test at 24 h after ICH. Data were represented as mean ± SD. # *p* < 0.05 vs Naïve + Scr siRNA, $ *p* < 0.05 vs ICH + Scr siRNA. One-way ANOVA, Tukey test, *n* = 6/group

To validate the knockdown efficacy of NLRC4 siRNA, western blot was conducted to detect protein expression in both naïve and ICH animals. NLRC4 siRNA administered via i.c.v injection significantly reduced NLRC4 expression in both naïve and ICH mice (Fig. 7A, B). Consistently, the expression of C-Caspase-1 also decreased, and ICH-induced neurological dysfunction was ameliorated by NLRC4 knockdown in vivo (Fig. 7C–F).

### The NK1R agonist, GR73632, and PKCδ activator, PMA, reversed the neuroprotective effects of Aprepitant at 24 h after ICH

**Fig. 8:**
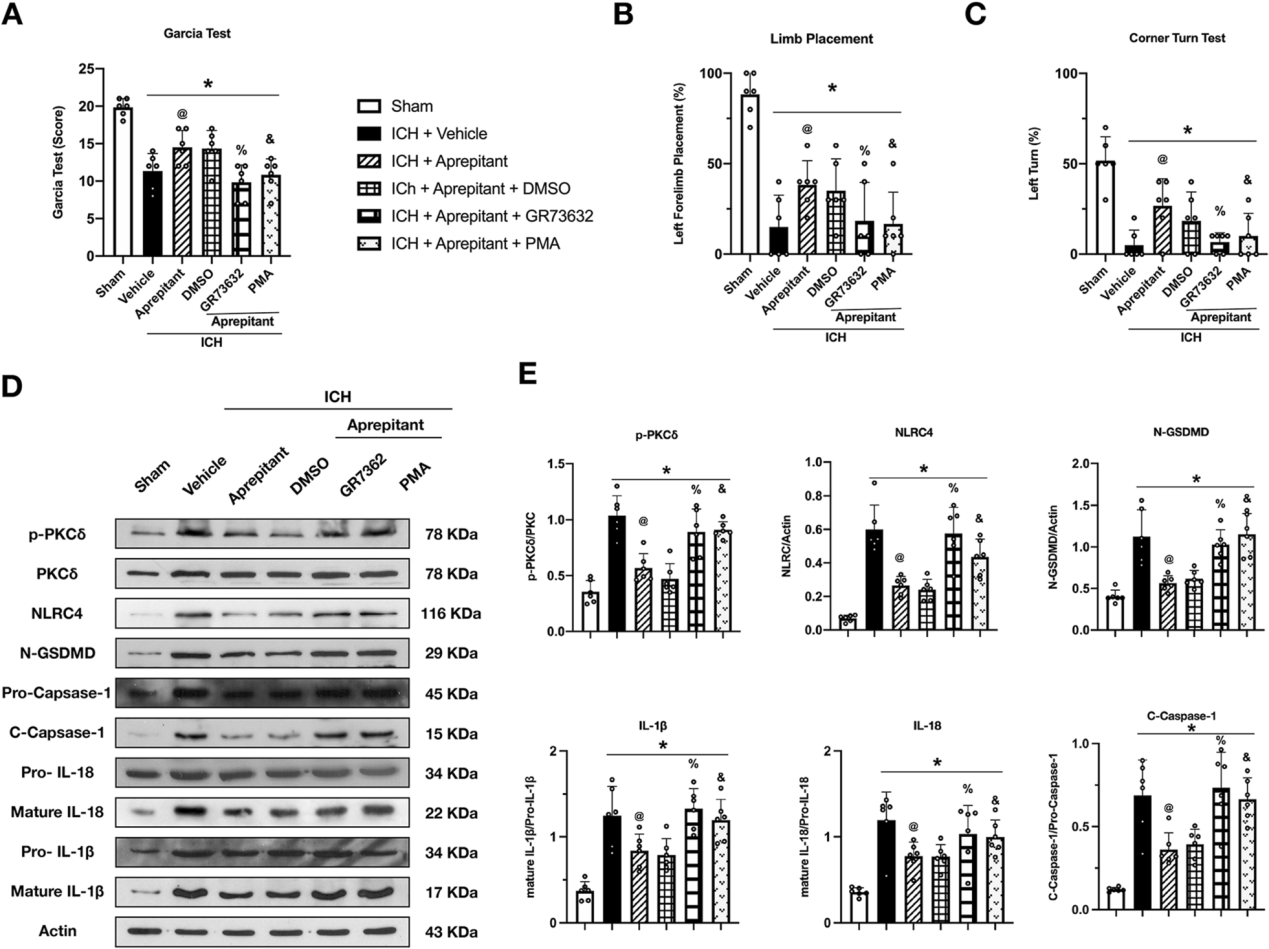
Activation of NK1R or PKCδ abolished the effects of Aprepitant after ICH. **A**–**C** Modified Garcia test, forelimb placement test, and corner turn test at 24 h after ICH. **D** Representative western blot bands, **E** quantitative analyses of p-PKCδ, NLRC4, N-GSDMD, C-Caspase-1, IL-18, and IL-1β at 24 h after ICH. Data were represented as mean ± SD. * *p* < 0.05 vs Sham, @ *p* < 0.05 vs ICH + vehicle, % *p* < 0.05 vs ICH + Aprepitant + DMSO; & *p* < 0.05 vs ICH + Aprepitant + DMSO; one-way ANOVA, Tukey post hoc test, *n* = 6/group

To further explore possible downstream signaling pathways, an NK1R selective agonist, GR73632, and PKC agonist, PMA, were selected for mechanism studies. It was found that GR73632 and PMA could reverse the neuroprotective effects of Aprepitant treatment at 24 h after ICH in the modified Garcia test (Fig. 8A), forelimb placement test (Fig. 8B), and corner turn test (Fig. 8C). Western blot results showed that the expression of p-PKC, NLRC4, C-Caspase-1, N-GSDMD IL-18, and IL-1β was significantly increased at 24 h after ICH compared with the sham group. However, the expression of these proteins was decreased in the ICH + Aprepitant group compared with the ICH + vehicle group. GR73632, a selected agonist of NK1R, and PKC agonist, PMA, were administered at 1 h after ICH to assess the mechanism of Aprepitant in the antioxidant stress and anti-pyroptosis pathways after ICH. Our data showed that treatment with GR73632 and PMA significantly increased the expression of p-PKC, NLRC4, Romo-1, C-Caspase-1, and IL-1β compared with the ICH + Aprepitant + DMSO group (Fig. 8D, E). These results suggest that Aprepitant attenuates oxidative stress and NLRC4-dependent neuronal pyroptosis via NK1R/PKCδ pathway after intracerebral hemorrhage.

## Discussion

In the present study, we explored the effects of NK1R inhibition with Aprepitant on neuronal pyroptosis in a mouse model of ICH, and we investigated possible underlying mechanisms. We found that the protein levels of NK1R and NLRC4 increased in a time-dependent manner after ICH, and peaked at 24 h after ICH. NK1R co-localized with neurons. In addition, treatment with Aprepitant significantly improved neurological function, attenuated neuronal pyroptosis, and reduced the expression of p-PKCδ, NLRC4, cleaved caspase-1, IL-1β, and IL-18 24 hours after ICH. In contrast, activating NK1R with the selective agonist, GR73632, or using the agonist of PKCδ, PMA, reversed the neuroprotective effects of Aprepitant. In addition, knockdown of NLRC4 by siRNA produced similar effects to Aprepitant treatment, including improved neurological outcomes, as well as downregulation of NLRC4 and cleaved caspase-1 expression after ICH. These observations suggest that Aprepitant reduces NLRC4-dependent neuronal pyroptosis after ICH, possibly through inhibition of the NK1R / PKCδ signaling pathway.

Recently, pyroptosis, and its relationship with inflammation, has attracted growing attention in the study of cerebral ischemia or hemorrhage [12]. Pyroptosis is an inflammatory form of programmed cell death (PCD) involving activation of caspase-1 by inflammasomes [37]. Activated caspase-1 processes pro-IL-1β and pro-IL-18 into the mature inflammatory cytokines, IL-1β and IL-18, respectively, while cleaves Gasdermin D (GSDMD). The N-terminal fragment of GSDMD assembles into a plasma membrane pore in the cell membrane, from which bioactive IL-1β and IL-18, as well as other cellular contents, are subsequently released to induce inflammation and cause cell death [37–39]. Therefore, inflammasomes are an essential and important component in the entire process of pyroptosis. Unlike inflammasomes, such as NLRP1 and NLRP3, apoptosis-associated speck-like protein containing CARD (ASC) is not essential for the assembly of the NLRC4 inflammasome [12, 40]. NLRC4 and pro-caspase-1 can directly assemble into an inflammasome through their CARD (C-terminal caspase activating and recruitment domain) interactions, but interestingly, the presence of ASC promotes the secretion of IL-1β and IL-18 [41]. Poh et al. showed through in vivo and in vitro experiments that the NLRC4 inflammasome mediates the inflammatory response, as well as microglial apoptosis and pyroptosis in the ischemic stroke model [42]. Conversely, a study by Sui et al. confirmed a significant increase in NLRC4 expression in TNA2 astrocytes in the rat brain after cerebral ischemia [43]. An additional study also confirmed that after cerebral ischemia onset, NLRC4 expression was significantly upregulated and localized mainly in neurons [44]. Regarding NLRC4, there are limited data regarding its role after ICH. Durocher et al. found significant upregulation of the NLRC4 gene in peripheral blood from patients by whole-transcriptome analysis of peripheral blood from patients with ICH [45]. Consistently, we observed a time-dependent upregulation of endogenous expression of the NLRC4 inflammasome in the right hemisphere after ICH, which started at 3 h and peaked at 24 h after ICH. To further examine the role played by NLRC4 in ICH, we knocked down the protein level of NLRC4 using siRNA, and showed that mice in the ICH + NLRC4 siRNA group had a better neurological outcome compared to the control group, as well as a decrease in the expression of C-Caspase-1. These exciting results suggest that targeting NLRC4 may a very promising direction for the treatment of ICH.

NK1R is widely distributed in the central nervous system [46]. In 1996, Andoh et al. identified the presence of NK1R mRNA in the olfactory bulb, cerebral cortex, medulla oblongata, and spinal cord of rats [47]. In 2003, Caberlotto et al. showed that NK1R was highly expressed in the cerebral cortex and ventral striatum, whereas there was reduced expression in the hippocampus and amygdala [46]. Conversely, previous studies have shown that NK1R is expressed in neurons, microglia, astrocytes, and other immune cells [48]. Similarly, we confirmed that NK1R is abundantly expressed in neurons after ICH by double immunofluorescence. In addition, previous studies found that NK1R expression was significantly elevated after brain injury in a traumatic brain injury model [49]. Consistent with these findings, we found that NK1R expression was significantly increased after ICH and peaked at 24 h after ICH.

The concept that inhibition of NK1R can exert neuroprotective effects is not a new one. Inhibiting NK1R exerted a neuroprotective effect in various neurological disease models, such as traumatic brain injury, cerebral infarction, encephalitis, and subarachnoid hemorrhage [36, 50–52]. Our previous study also found that inhibition of NK1R improved the neurological outcome by promoting hematoma clearance through modulation of microglial polarization after ICH [20]. As research on NK1R progressed, a number of NK1R-specific inhibitors were developed. To facilitate translation of our findings into clinical application, we chose Aprepitant, the first FDA-approved NK1R-specific antagonist available for clinical use, is used primarily for the treatment of chemotherapy-induced nausea and vomiting [53]. The benefit of administering drugs intraperitoneally is the ability for the peritoneal cavity to absorb large amounts of a drug quickly. Therefore, in our study, as in other studies related to Aprepitant [36, 54], intraperitoneal injection was selected as a more suitable method of administration. One of the factors that limit the use of drugs to treat central nervous system disorders is related to the ability of the drug to cross the blood–brain barrier (BBB). Studies have confirmed that Aprepitant can cross the blood–brain barrier, making it possible to treat neurological disorders [53, 55]. However, the effect of Aprepitant on neuronal pyroptosis has not been elucidated. In this study, we found that Aprepitant treatment significantly improved neurological dysfunction and inhibited the expression of NLRC4 inflammasome, cleaved caspase-1, IL-1β, N-GSDMD, and IL-18 after ICH. Importantly, the results of the immunofluorescence experiments showed that Aprepitant reduced the number of degenerating neurons, especially cleaved caspase-1-positive neurons after ICH, suggesting that Aprepitant inhibited NLRC4 inflammasome-mediated pyroptosis. Thus, inhibition of the anti-pyroptotic death properties of NK1R receptors with Aprepitant may have neuroprotective effects in ICH. In addition, previous studies have found that the hippocampal CA1 region is prone to secondary cerebral ischemia after ICH [8, 56], moreover, after ICH, the unabsorbed red blood cells in the hematoma lyse, releasing potentially harmful components such as hemoglobin and iron into the extracellular space. These harmful substances may in turn affect the survival of hippocampal neurons through the circulation of cerebrospinal fluid [57]. And the neurons in the CA1 region, which is mainly associated with learning and memory functions, are very sensitive to this. Therefore, animals often exhibit learning and memory dysfunction after cerebral hemorrhage [31]. The results of the Morris water maze test showed that Aprepitant treatment significantly improved long-term memory and cognitive impairment in ICH mice, and further FJC staining showed that neuronal degeneration in the hippocampal CA1 region was significantly attenuated in the treated mice. These results suggest that early inhibition of SP/NK1R signaling can provide long-term neuronal protection and improve sensorimotor and cognitive dysfunction in ICH mice.

The binding of SP to NK1R activates phospholipase C (PLC), which leads to the formation of inositol trisphosphate and diacylglycerol. Inositol trisphosphate increases intracellular Ca^2+^ levels, whereas diacylglycerol activates protein kinase C (PKC) [21]. Koh et al. showed that SP/NK1R activation significantly upregulated PKC-α and PKC-δ expression [22]. The study by Wang et al. also found that PKCδ inhibitors significantly reduced SP/NK1R-mediated NOX2 activation [23]. These results suggest that PKCδ may be one of the major downstream signaling pathways of SP/NK1R. Moreover, PKCδ is one of the major upstream regulatory proteins of NLRC4 [25, 58]. A study by Samidurai et al. found that TWEAK-induced PKCδ activation enhances the NLRC4 signaling pathway [24]. In our present study, we likewise found that inhibition of SP/NK1R using Aprepitant resulted in a significant reduction in the phosphorylation level of PKCδ, which subsequently induced a decrease in NLRC4 expression, as well as a downregulation of cleaved caspase-1, N-GSDMD, IL-1β, and IL-18.

To further validate this possible underlying mechanism, we administered the selective NK1R receptor agonist, GR73632, and PMA, a PKCδ selective agonist, concomitantly with Aprepitant treatment. They abolished the effects of Aprepitant on neurological improvement and reversed the inhibitory effects of Aprepitant on downstream proteins, including PKCδ, NLRC4 inflammasome, cleaved caspase-1, N-GSDMD, IL-1β, IL-18. Similarly, when siRNA was administered to knock down NLRC4 prior to ICH induction, it produced similar neuroprotective effects and inhibited cleaved caspase-1 in our ICH model. Thus, these observations suggest that intraperitoneal injections of Aprepitant produce neuroprotective effects that may inhibit NLRC4-dependent neuronal pyroptosis by suppressing the NK1R/PKCδ signaling pathway after ICH.

There are some limitations of the present study. First, previous studies have reported that inhibition of NK1R receptors exerts neuroprotective effects through multiple mechanisms, including blood–brain barrier protection, anti-apoptosis, anti-oxidation, and regulation of microglia polarization [20, 51]. In this study, we focused on the anti-pyroptotic effects of NK1R receptors after ICH. Therefore, further elucidation of other neuroprotective mechanisms of NK1R receptor inhibition after ICH is needed. Second, since NLRC4 is mainly expressed in neurons [44], this inflammasome may be more closely associated with neuronal pyroptosis, but we cannot exclude the possibility of other inflammasomes including NLRP1 and NLRP3 in neuronal pyroptosis [12]. Third, our previous study found that NK1R is expressed not only on neurons, but also on glial cells [59]. Therefore, it is possible that Aprepitant also exerts neuronal protective effects through glial cell–neuron crosstalk, for example, by reducing neuroinflammation and maintaining the integrity of the BBB. Therefore, in the next study, we would like to fully elaborate the protective role of Aprepitant in neurons through in vitro experiments.

## Conclusions

The present study shows that Aprepitant inhibits NLRC4-dependent neuronal pyroptosis and neurological deficits after experimental ICH in mice at least partially through the NK1R/PKCδ signaling pathway. The use of Aprepitant, a specific inhibitor of NK1R, may provide a promising therapeutic strategy for the treatment of patients with ICH.

## Supplementary Information

**Additional file 1. **Changes in protein expression of NK1R in the hippocampal region after ICH.

## Data Availability

The data support the findings of this study and are available from the corresponding author upon reasonable request.

## Abbreviations

AD: Alzheimer's disease
AIM2: Absent In Melanoma 2
CNS: Central nervous system
CREB: Cyclic adenosine monophosphate response element-binding
DAG: Diacylglycerol
EAE: Experimental autoimmune encephalomyelitis
FJC: Fluoro-Jade C
GSDMD: Gasdermin D
GPCR: G protein-coupled receptor
IACUC: Institutional Animal Care and Use Committee
ICH: Intracerebral hemorrhage
IP3: Inositol 1,4,5 trisphosphate
NK1R: Neurokinin receptor 1
NLRP1: NLR Family Pyrin Domain Containing 1
NLRP3: NLR Family Pyrin Domain Containing 3
NLRC4: NLR Family CARD Domain Containing 4
PLC: Phospholipase C
PKCδ: Protein kinase C delta
PMA: Phorbol 12-myristate 13-acetate
siRNA: Small interfering ribonucleic acid
SBI: Secondary brain injury
SP: Substance P
TUNEL: Terminal deoxynucleotidyl transferase dUTP nick end labeling
