# Supplementary material for: Aprepitant attenuates NLRC4-dependent neuronal pyroptosis via NK1R/PKCδ pathway in a mouse model of intracerebral hemorrhage
Source: J Neuroinflammation. 2022 Aug 3;19:198. doi: 10.1186/s12974-022-02558-z (PMC9351153; doi:10.1186/s12974-022-02558-z)
Supplement: Supplementary file 1 — Additional file 1. Changes in protein expression of NK1R in the hippocampal region after ICH. [file 12974_2022_2558_MOESM1_ESM.docx]

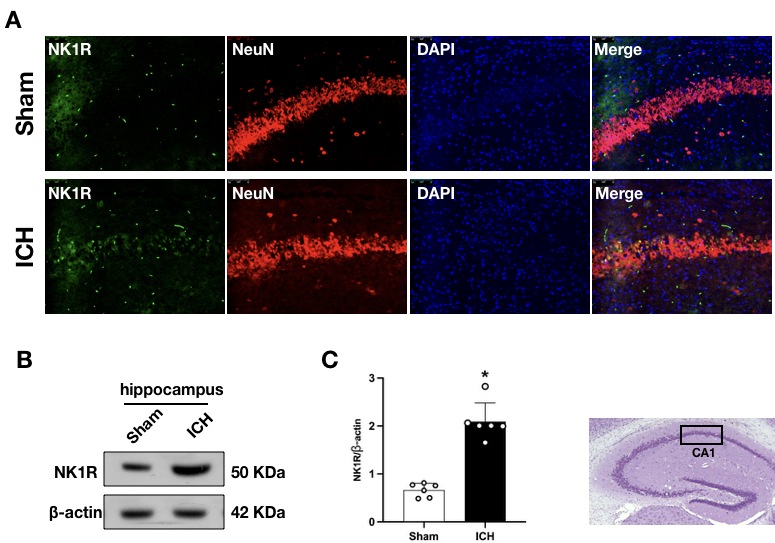


**Fig. S1 Changes in protein expression of NK1R in the hippocampal region after ICH.** (A) Representative pictures of double immunofluorescence staining showed NK1R (green) colocalized with neurons (NeuN, red) in CA1 region of hippocampal 24 h after ICH. (B) Representative western blot bands, and (C) quantitative analyses of NK1R in the hippocampal region after ICH. Data was represented as mean ± SD. *p < 0.05 vs sham group; One-way ANOVA, Tukey test, n=6/group.
